# Determinants of undesired α2-6-sialoside formation by PmST1 M144D

**DOI:** 10.1039/d5ob01796c

**Published:** 2025-12-29

**Authors:** Fahima Mozaneh, Maju Joe, Warren W. Wakarchuk, Peng Wu, Matthew S. Macauley

**Affiliations:** a Department of Chemistry, University of Alberta Edmonton Canada T6G 2G2 macauley@ualberta.ca; b Department of Biological Sciences, University of Alberta Edmonton Canada T6G 2E9; c Department of Molecular Medicine, The Scripps Research Institute La Jolla California 92037 USA; d Department of Medical Microbiology and Immunology, University of Alberta Edmonton Canada T6G 2E1

## Abstract

Sialyltransferases catalyze regioselective glycosidic bond formation between sialic acid and a glycan acceptor. *Pasteurella multocida* α2-3-sialyltransferase 1 (PmST1) is a widely used enzyme in chemoenzymatic synthesis. In particular, the PmST1 M144D mutant is routinely employed as an α2-3-sialyltransferase, although only low levels of α2-6-sialyltransferase activity have been reported. Here, we discover that for certain acceptors, the formation of the undesired α2-6-sialoside can reach up to 20% of the product. To elucidate the factors that influence this regioselectivity, we systematically examined the effects of (i) sulfation of the acceptor, (ii) the chemical nature of the aglycone, (iii) pH, and (iv) the extent of reaction completion. The results indicate that sulfation at the 6-position of GlcNAc or a β-ethyl-NHCbz aglycone is a factor that can increase the amount of α2-6 sialoside product. Surprisingly, pH had only a small impact, and the amount of α2-6 sialoside product did not differ over the course of the reaction. These findings provide insights into the enzymatic specificity of PmST1 M144D and inform its optimized use in chemo-enzymatic synthesis of defined sialosides.

## Abstract 2

Regioselectivity of PmST1 M144D as influenced by different factors.

## Graphical abstract

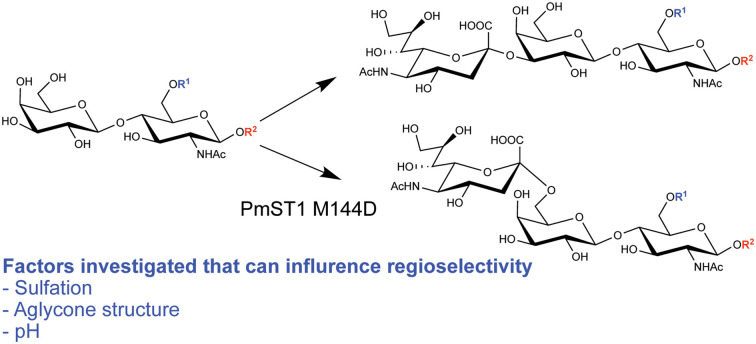

## Introduction

Sialyltransferases are a class of glycosyltransferases that catalyze the transfer of a sialic acid residue to the terminal position of oligosaccharides on glycoproteins, glycolipids, or free oligosaccharides.^1–3^ Sialyltransferases use an activated donor as their source of sialic acid in the form of cytidine-5′-monophospho-sialic acid (CMP-sialic acid). According to the Carbohydrate-Active enZYmes (CAZy) database, all eukaryotic sialyltransferases are classified into a single glycosyltransferase family, GT29.^4,5^ In contrast, bacterial sialyltransferases are categorized into four CAZy glycosyltransferase families: GT38, GT42, GT52, and GT80 (https://www.cazy.org).

*Campylobacter jejuni* (Cst-l) from the GT42 family and *Pasteurella multocida* (PmST1) are GT80 family members routinely used in chemoenzymatic synthesis.^6^ PmST1 has seen widespread use in chemoenzymatic synthesis due to its desirable properties that include high expression levels in *E.* coli (100 mg L^−1^ culture), high catalytic activity, promiscuity toward a wide range of acceptors and modified CMP-sialic acid donors,^6–12^ and activity towards acceptors on the cell surface.^13^ PmST1 was initially characterized as an α2-3-sialyltransferase, exhibiting activity across a broad pH range (6.0–10.0) with an optimal pH range between 7.5 and 9.0. In addition to its main function, it displays secondary enzymatic activities, including weak α2-6-sialyltransferase activity at pH < 7, α2-3-sialidase activity at pH values of 5.0–5.5 (effectively the back reaction), and α2-3-*trans*-sialidase activity at pH values of 5.5–6.5.^6,7,10,14,15^ The α2-6-sialyltransferase activity of WT PmST1 was reported to be minimal at pH values above 7.5.^8^ However, the donor hydrolysis and α2-3-sialidase activities of PmST1 can significantly reduce the efficiency of sialyltransferase-catalyzed reactions, resulting in low product yields. To address this limitation, the PmST1 M144D mutant was engineered to reduce the α2-3 sialidase and donor hydrolysis activities.^14^ Other advantages of PmST1 M144D are that it is used as a versatile enzyme for synthesizing more complicated trisaccharides with acceptors that are not necessarily on the non-reducing end of the carbohydrate.^16^

In WT PmST1, deep donor binding induces closure of the active site and positions Trp270 to shape a well-defined acceptor pocket, enforcing specific acceptor orientations that favor either α2-3 or α2-6 linkage formation.^14,17^ In the M144D mutant, the donor binding is shallower in the active site, preventing Trp270 from moving into place and leaving the acceptor region less organized. We speculate that such differences in the active site architecture could cause the mutant to have different regioselectivity depending on the nature of the acceptor.

In this study, PmST1 M144D-catalyzed sialylation reactions were performed using a variety of acceptors and CMP-Neu5Ac as the donor. We discovered that PmST1 M144D can generate the undesired α2-6 sialoside product to significant extents when certain acceptors are used, even under basic conditions. This observation led us to investigate the variables that influence the ratio of the desired α2-3 sialoside products *versus* the undesired α2-6 sialoside product. Specifically, sulfation of the underlying GlcNAc residue and a certain aglycone structure, such as β-ethyl-NHCbz, can significantly enhance the undesired α2-6 sialoside product. Reactions were analyzed across different extents of completion and pH values, with basic pH only showing a modest decrease in the undesired α2-6 product. Notably, CST-06, a version of Cst-I that is a fusion protein with maltose binding protein,^18,19^ and human ST3Gal4 did not show any α2-6-linked product under any conditions tested. Overall, our findings caution that chemoenzymatic reactions with PmST1 M144D require purification to remove significant amounts of the α2-6-linked product for certain acceptors, with CST-06 being a suitable alternative in these cases.

## Results and discussion

### PmST1 M144D activity on *O*-sulfated and non-*O*-sulfated disaccharides

**Scheme 1:**
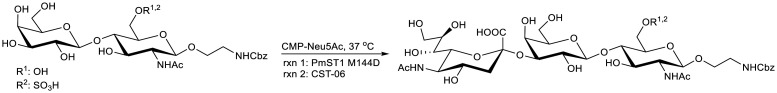
Enzymatic synthesis of sulfated and non-sulfated Neu5Ac-α2-3-LacNAc-β-ethyl-NHCbz. Reagents and conditions: CMP-Neu5Ac (2 eq.), 100 mM Tris-HCl (pH 8.5), 20 mM MgSO_4_, α2-3-sialyltransferases (PmST1 M144D and CST-06), and 37 °C.

**Fig. 1:**
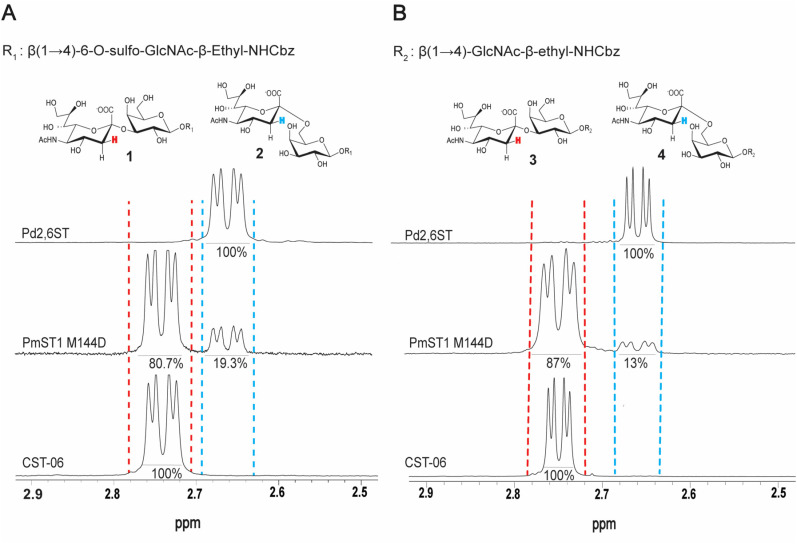
^1^H NMR analysis of product formation using different sialyltransferases. (A) The bottom spectrum shows the control compound Neu5Ac-α2-3-Gal-β(1 → 4)-6-*O*-sulfo-GlcNAc-β-ethyl-NHCbz, enzymatically synthesized using CST-06. The middle spectrum displays the product mixture obtained from a sialylation reaction catalyzed by PmST1 M144D, which contains both α2-3- and α2-6-linked sialylated products. The top spectrum shows the control compound Neu5Ac-α2-6-Gal-β(1 → 4)-6-*O*-sulfo-GlcNAc-β-ethyl-NHCbz, synthesized enzymatically using Pd2,6ST. (B) The bottom spectrum shows the control compound Neu5Ac-α2-3-LacNAc-β-ethyl-NHCbz, enzymatically synthesized using CST-06. The middle spectrum displays the product mixture obtained from a sialylation reaction catalyzed by PmST1 M144D, which contains both α2-3- and α2-6-linked sialylated products. The top spectrum shows the control compound Neu5Ac-α2-6-LacNAc-β-ethyl-NHCbz, synthesized enzymatically using Pd2,6ST.

We previously used PmST1 M144D for the synthesis of a series of sulfated sialosides on a LacNAc-β-ethyl-NH_2_ core, which were purified by HPLC.^20^ To expedite the purification process, we began using size-exclusion chromatography (SEC; P-2 and LH-20 resins) to purify the Neu5Ac-α2-3-Gal-β(1 → 4)-6-*O*-sulfo-GlcNAc-β-ethyl-NHCbz product (Scheme 1). The β-ethyl-NHCbz aglycone was used to have an orthogonal protecting group for potential use in combination with azido sugars. These reactions went to completion and were readily cleaned up from other products (*e.g.* excess CMP-sialic acid and CTP) by SEC. However, upon close analysis of the product by proton nuclear magnetic resonance (^1^H NMR) spectroscopy, two sets of peaks were apparent for the H3 chemical shifts (Fig. 1A), with only one mass observed by high resolution mass spectrometry (Fig. S1, SI). In this experiment and the subsequent ones, the ^1^H NMR spectra of all PmST1 M144D-catalyzed reactions were obtained after purification by SEC and were zoomed in on the H_3 eq._ region to clearly resolve and distinguish the distinct peaks corresponding to the different chemical shifts (ppm) of α2-3- and α2-6-linked sialosides. Importantly, comparison with the acquired spectra of CMP-Neu5Ac and free Neu5Ac (Fig. S10, SI) confirmed that the additional peaks did not arise from these species; their chemical shifts were consistent only with the formation of the α2-6-linked sialoside. The extent of the α2-6-linked sialylated product was 19.3 ± 1.3% over four independent experiments. These results were observed despite using a pH of 8.5 in the enzymatic assay, which was reported to limit the amount of the α2-6 sialoside product using WT PmST1.^8^ Parallel reactions were performed using two other sialyltransferases: CST-06 α2-3-sialyltransferase and *Photobacterium damselae* α2-6-sialyltransferase (Pd2,6ST). The products of these reactions were purified in the same manner as above, and only one set of H3 chemical shifts was observed with the anticipated linkage (Fig. 1A). We were curious if the significant amount of α2-6 product was due to the 6-*O*-sulfation in the acceptor; therefore, we repeated the experiments on non-sulfated LacNAc-β-ethyl-NHCbz and in this case the percentage of the α2-6 product was 13.0 ± 1.0% over four independent experiments (Fig. 1B). These findings indicate that the presence of a sulfate group on the acceptor does influence the regioselectivity of PmST1 M144D, leading to the formation of more of the undesirable α2-6 product. Therefore, we went on to examine additional parameters governing the regioselectivity of PmST1 M144D.

### Influence of the aglycone structure on PmST1 M144D regioselectivity

**Fig. 2:**
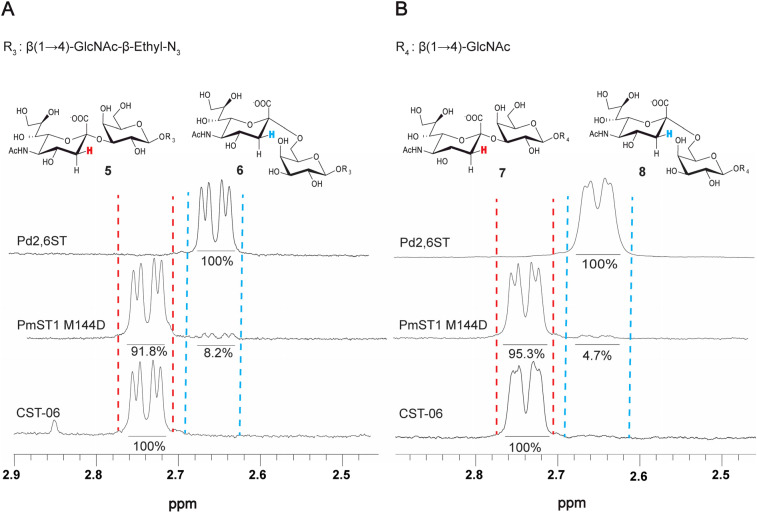
^1^H NMR analysis of sialylated products with acceptors bearing different aglycones. (A) The bottom spectrum shows the control compound Neu5Ac-α2-3-LacNAc-β-ethyl-N_3_ enzymatically synthesized using CST-06. The middle spectrum displays the product mixture obtained from a sialylation reaction catalyzed by PmST1 M144D, which contains both α2-3- and α2-6-linked sialylated products. The top spectrum shows the control compound Neu5Ac-α2-6-LacNAc-β-ethyl-N_3_, synthesized enzymatically using Pd2,6ST. (B) The bottom spectrum shows the control compound Neu5Ac-α2-3-LacNAc, enzymatically synthesized using CST-06. The middle spectrum displays the product mixture obtained from a sialylation reaction catalyzed by PmST1 M144D, which contains both α2-3- and α2-6-linked sialylated products. The top spectrum shows the control compound Neu5Acα2-6LacNAc, synthesized enzymatically using Pd2,6ST.

To investigate the influence of the aglycone structure on the regioselectivity of PmST1 M144D, two additional acceptors were investigated with different groups at the anomeric center: LacNAc in its reducing form and LacNAc-β-ethyl-N_3_. As before, the trisaccharide products were analyzed by ^1^H NMR for the H_3 eq._ chemical shifts in the Neu5Ac of the trisaccharide products as a readout of α2-3 and α2-6 products. LacNAc-β-ethyl-N_3_ averaged 8.2 ± 0.2% of the α2-6 product over three independent experiments, while LacNAc averaged 4.7% ± 0.6% over three independent experiments (Fig. 2A and B). This observation suggests that the β-ethyl-NHCbz aglycone, shown in Fig. 1B, negatively influenced PmST1 M144D regioselectivity as it had the highest amount of α2-6 product. The increased formation of the α2-6-linked product in the presence of the bulky NHCbz group may be attributed to steric effects introduced by the larger, more hydrophobic carbobenzyloxy group, which could alter substrate orientation within the enzyme's active site. In summary, structural variations at the aglycone (reducing end) can significantly modulate the regioselectivity of PmST1 M144D, with bulkier groups favouring increased formation of the undesired α2-6 product.

### Influence of pH on PmST1 M144D regioselectivity

**Fig. 3:**
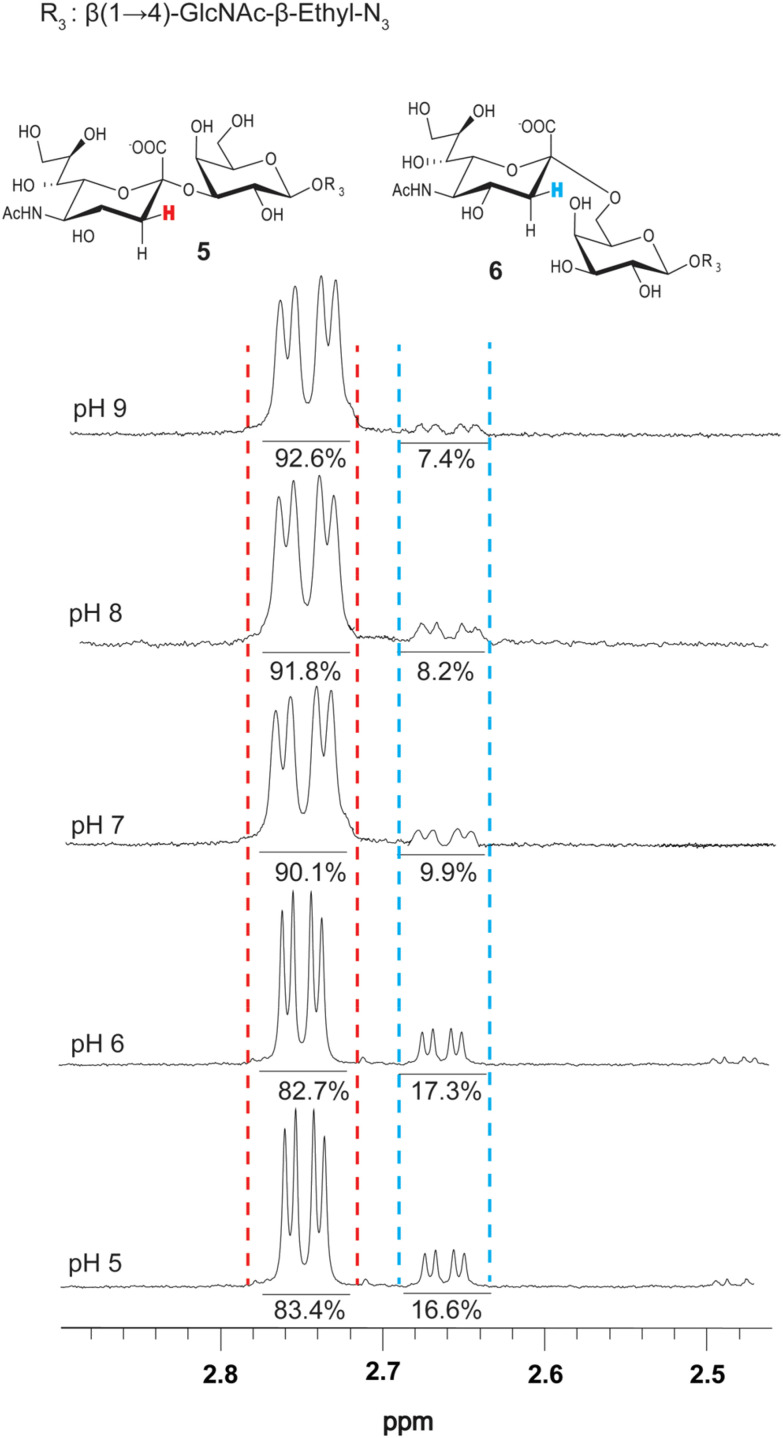
^1^H NMR analysis of PmST1 M144D-catalyzed sialylation of LacNAc-β-ethyl-N_3_ across a pH range of 5.0 to 9.0. Formation of desired and undesired sialylated products, including Neu5Acα2-6LacNAc-β-ethyl-N_3_, with pH-dependent changes in α2-6-sialylation peak intensities.

Previously, it was reported that the α2-6-sialyltransferase activity of WT PmST1 is significantly reduced at pH values above 7.^8^ Despite using a pH of 8.5 in all experiments described above, which should have minimized the α2-6 product, we still felt that it was warranted to investigate how pH influences PmST1 M144D regioselectivity. Therefore, a series of reactions were conducted across a pH range of 5.0 to 9.0 using LacNAc-β-ethyl-N_3_ as the acceptor. Product formation was monitored over a 15 minute reaction time, and all reactions reached completion at every pH. Product formation was analyzed by ^1^H NMR, as described above, to quantify the percentage of the α2-6-sialylated product (Fig. 3). The formation of Neu5Ac-α2-6-LacNAc-β-ethyl-N_3_ decreased progressively with increasing pH but remained detectable at pH 9. Specifically, the average values of the α2-6 product were 16.6 ± 0.5% at pH 5.0, 17.3 ± 0.9% at pH 6.0, 9.9 ± 0.6% at pH 7.0, 8.2 ± 0.2% at pH 8.0, and 7.4 ± 0.1% at pH 9.0 over three independent reactions at each pH. These findings indicate that while α2-6-sialyltransferase activity is pH-sensitive and diminished under basic conditions, PmST1 M144D retains significant residual catalytic capacity for α2-6-linkage formation across a broad pH range. These results contrast with results reported with WT PmST1, where there was almost complete loss of α2-6-activity above pH 7.^8^

### Regioselectivity of PmST1 M144D as a function of reaction completion

**Fig. 4:**
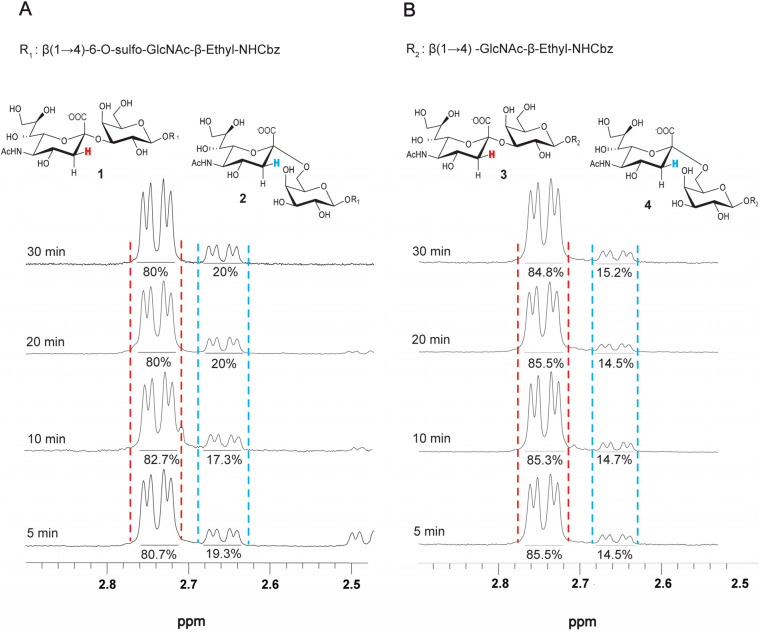
Time-course study of PmST1 M144D-catalyzed sialylation. (A) ^1^H NMR analysis of H_3 eq._ of Neu5Ac spectra showing the integration of α2-3- and α2-6-sialylation reactions catalyzed by PmST1 M144D using Gal-β(1 → 4)-6-*O*-sulfo-GlcNAc-β-ethyl-NHCbz as the acceptor substrate in the time course of 5 to 30 minutes. (B) ^1^H NMR analysis of H_3 eq._ of Neu5Ac spectra showing the integration of α2-3- and α2-6-sialylation reaction catalyzed by PmST1 M144D using LacNAc-β-ethyl-NHCbz as the acceptor substrate in the time course of 5 to 30 minutes.

WT PmST1 possesses both sialidase and *trans*-sialidase activities, which are markedly reduced in the M144D mutant. Given that the benefits of suppressing these side activities in PmST1 M144D outweigh the drawback of slower sialylation kinetics,^21^ we sought to confirm that any residual activities did not influence the outcomes of reactions allowed to proceed to completion. Therefore, a series of sialylation reactions were conducted over varying time intervals. The reactions employed LacNAc-β-ethyl-NHCbz and Gal-β(1 → 4)-6-*O*-sulfo-GlcNAc-β-ethyl-NHCbz as acceptors. The reactions were stopped at different time points, the reaction mixtures were purified by SEC, and product purity was assessed by ^1^H NMR for the ratio of α2-3 and α2-6 products (Fig. 4A and B). After five minutes, approximately 25% of the total reaction had occurred, while the reaction was approximately 50% and 75% complete at ten and twenty minutes, respectively, and by 30 minutes, the reaction had gone to completion. The results show that the percentage of the α2-6 product was relatively constant throughout the course of the reaction. These results rule out any confounding effects from sialidase activity and demonstrate that the α2-3 and α2-6 products are formed with similar reaction rates.

## Conclusions

In conclusion, we have demonstrated that the structure of the acceptors significantly affects the ratio of α2-3- to α2-6-sialylated products formed by PmST1 M144D. Specifically, Gal-β(1 → 4)-6-*O*-sulfo-GlcNAc-β-ethyl-NHCbz led to a higher proportion of α2-6-sialylated products compared to the non-sulfated LacNAc-β-ethyl-NHCbz. Among the tested aglycone variants, LacNAc with β-ethyl-NHCbz resulted in a higher α2-6-sialylation ratio than both β-ethyl-N_3_ and LacNAc without an aglycone. Although higher pH levels reduce the overall percentage of α2-6-sialylated products, they do not mitigate the α2-6-sialyltransferase activity of PmST1 M144D.

## Author contributions

F. M.: writing – original draft, conceptualization, data curation, investigation, validation, and visualization. M. J.: resources and writing – review & editing. W. W.: resources and writing – review & editing. P. W.: resources and writing – review & editing. M. S. M.: conceptualization, funding acquisition, methodology, project administration, resources, supervision, validation, writing – original draft, and writing – review & editing.

## Conflicts of interest

There are no conflicts to declare.

## Supplementary Material

OB-024-D5OB01796C-s001

## Data Availability

The data supporting this article have been included as part of the supplementary information (SI). Supplementray information: general methods, synthetic procedures, characterisation data, and NMR spectra for all compounds. See DOI: https://doi.org/10.1039/d5ob01796c. Raw data underlying the study, including compound characterization and mass spectrometry raw chromatograms, are securely archived at the University of Alberta and are available upon request.
