# Supplementary material for: Determinants of undesired α2-6-sialoside formation by PmST1 M144D
Source: Org Biomol Chem. 2025 Dec 29;24(5):1027–32. doi: 10.1039/d5ob01796c (PMC12797295; doi:10.1039/d5ob01796c)
Supplement: OB-024-D5OB01796C-s001 [file OB-024-D5OB01796C-s001.pdf]

This journal is © The Royal Society of Chemistry 2026

Supporting Information

Determinants of undesired α2-6-sialoside formation by PmST1 M144D

Fahima Mozaneh[1], Maju Joe[1], Warren W. Wakarchuk[2], Peng Wu[3], Matthew S. Macauley[1,4]

[1]Department of Chemistry, University of Alberta, Edmonton, Canada, T6G 2G2

[2] Department of Biological Sciences, University of Alberta, Edmonton, Canada T6G 2E9

[3]Department of Molecular Medicine, The Scripps Research Institute, La Jolla, California 92037, United States

[4]Department of Medical Microbiology and Immunology, University of Alberta, Edmonton, Canada T6G 2E1

Table of Contents

1. General Information……………………………………………………………………………S2
2. Procedure for synthesis of α2-3 sialoside compounds **1-4**……..……………………………S3
3. Procedure for synthesis of α2-6 sialoside compounds **5-8**.....………………………………S3
4. References…………………………………………………………………………………...S4
5. Characterization Data…………………..………………………………………………….....S4
6. Copies of ^{1}H NMR and mass spectra...S6

General Information: All reagents and solvents were purchased from Sigma-Aldrich, Canada, and were used as received. $CDCl_3$ (chloroform D) and D_2O (deuterium oxide) were purchased from Deutero GmbH. Reactions were carried out in Eppendorf Flex-tubes. All reagents were purchased from commercial sources and were used without further. All reactions were carried out at 37 °C in shaking incubator and were monitored by TLC on Silica Gel 60 F_{254} (0.25 mm, E. Merck). Spots were detected under UV light (λ max = 254 nm) or by charring with 10% H_2SO_4 in EtOH. All column chromatography was performed on LH20. NMR experiments were conducted on a Varian 500 MHz instrument in the Chemistry NMR Facility, University of Alberta. For ^1H spectra, chemical shifts are expressed as parts per million (ppm, δ) and are relative to the solvent used. ^1H chemical shifts are reported relative to the deuterated solvent peak to HOD (4.78, D_2O). ^1H data were reported as though they were first order. Coupling constants (J) are reported in Hz and apparent multiplicities were described in standard abbreviations as singlet (s), doublet (d), doublet of doublets (dd), doublet of doublet of doublets (ddd), triplet (t), broad singlet (bs) and multiplet (m).

CMP-Neu5Ac (Cytidine-5′-monophospho-*N*-acetylneuraminic acid) Neu5Ac was purchased from Sigma Aldrich, and CMP-Neu5Ac was synthesized according to literature procedure (1).

Procedure for synthesis of α2-3 sialoside compounds

Acceptors (1 eq, 15 mmol) (Gal-β(1→4)-6-*O*-sulfo-GlcNAc-β-ethyl-NHCbz, LacNAc-β-ethyl-NHCbz, LacNAc-β-ethyl-N_3, and LacNAc) and CMP-Neu5Ac (2.0 eq) were dissolved in Tris-HCl buffer (pH 8.5) containing 20 mM $MgCl_2$. Recombinant shrimp alkaline phosphatase (rSAP, NEB; 3 μL) and CST-06 (0.1 mg/mL) were added to the reaction. The reaction tube was incubated overnight at 37 °C. Reaction progress was monitored by thin-layer chromatography (TLC) using a solvent system of iPrOH:NH_4OH:H_2O (7:2:1, v/v/v). Upon completion, reaction was quenched by a 4-fold dilution with cold absolute ethanol and incubated at −20 °C for 1 hour to precipitate the enzymes. The precipitated proteins were removed by centrifugation (3700 × g, 15 minutes, 4 °C). The resulting supernatant was carefully collected and evaporated under reduced pressure. The crude residues were resuspended in water and purified by size-exclusion chromatography on an LH20 column equilibrated with MeOH:H_2O (1:1), producing α2-3 sialoside compounds **1-4**.

Procedure for synthesis of α2-6 sialoside compounds

Acceptors (1 eq, 15 mmol) (Gal-β(1→4)-6-*O*-sulfo-GlcNAc-β-ethyl-NHCbz, LacNAc-β-ethyl-NHCbz, LacNAc-β-ethyl-N_3, and LacNAc) and CMP-Neu5Ac (2.0 eq) were dissolved in Tris-HCl buffer (pH 8.5) containing 20 mM $MgCl_2$. Pd2,6ST (0.1 mg/mL) were added to the reaction. The reaction tube was incubated overnight at 37 °C and the reaction progress was monitored by thin-layer chromatography (TLC) using a solvent system of iPrOH:NH_4OH:H_2O (7:2:1, v/v/v) (2). Upon completion, reaction was quenched by a 4-fold dilution with cold absolute ethanol and incubated at −20 °C for 1 hour to precipitate the enzymes. The precipitated proteins were removed by centrifugation (3700 × g, 15 minutes, 4 °C). The resulting supernatant was collected and evaporated under reduced pressure. The crude residues were resuspended in water and

purified by size-exclusion chromatography on an LH20 column equilibrated with MeOH:H_2O (1:1), producing α2-6 sialoside compounds**.**

References:

1. Yu H, Yu H, Karpel R, Chen X. Chemoenzymatic synthesis of CMP-sialic acid derivatives by a one-pot two-enzyme system: comparison of substrate flexibility of three microbial CMP-sialic acid synthetases. Bioorg Med Chem. 2004;12(24):6427–35.
2. Yu H, Chokhawala HA, Huang S, Chen X. One-pot three-enzyme chemoenzymatic approach to the synthesis of sialosides containing natural and non-natural functionalities. Nature Protocols. 2007;1(5):2485–92.

Characterization Data

2-(Benzyloxycarbonylaminoethyl)-5-acetamido-3,5-dideoxy-D-glycero-α-D-galacto-2-nonulopyranosylonate-(2→3)-β-D-galactopyranosyl-(1→4)-2-*N*-acetyl-2-deoxy-(6-*O*-sulfo)-β-D-glucopyranoside (1) (Neu5Ac-α2-3-Gal-6-*O*-sulfo-GlcNAc-β-Ethyl-NHCbz): white solid; yield 72%. ^{1}H-NMR (500 MHz, D_2O) δ = 7.36-7.48 (m, 5H), 5.16 (m, 2H), 4.59 (d, *J* = 8.0 Hz, 1H), 4.52 (d, *J* = 8.0 Hz, 1H), 4.39 (d, *J* = 11.0 Hz,1H), 4.30 (d, *J* = 9.5Hz, 1H) 4.11 (dd, *J* = 3, 10 Hz, 1H) 3.82-3.84 (m, 5H), 3.52-3.78 (m, 14H), 3.31 (bs, 2H), 2.75 (dd, *J* = 4.5, 12.5 Hz, 1H), 2.20 (s, 3H), 2.18 (s, 3H), 1.80 (t, *J* = 12 Hz, 1H).

2-(Benzyloxycarbonylaminoethyl)-5-acetamido-3,5-dideoxy-D-glycero-α-D-galacto-2-nonulopyranosylonate-(2→6)-β-D-galactopyranosyl-(1→4)-2-*N*-acetyl-2-deoxy-(6-*O*-sulfo)-β-D-glucopyranoside (2) (Neu5Ac-α2-6-Gal-β(1→4)-6-*O*-sulfo-GlcNAc-β-Ethyl-NHCbz): white solid; yield 73%. ^{1}H-NMR (500 MHz, D_2O) δ = 7.38-7.48 (m, 5H), 5.36 (m, 2H), 4.55 (d, *J* = 8.0 Hz, 1H), 4.46 (d, *J* = 8.0 Hz, 1H), 4.40 (d, *J* = 11.0 Hz,1H), 4.25 (dd, *J* = 5, 11.0Hz, 1H) 3.98 (t, J = 9.5 Hz, 1H), 3.85-3.94 (m, 4H), 3.75-3.84 (m, 3H), 3.61-3.75 (m, 9H), 3.49-3.58 (m, 3H), 3.31 (bs, 2H), 2.65 (dd, *J* = 5.0, 12.5 Hz, 1H), 2.10 (s, 3H), 1.98 (s, 3H), 1.70 (t, *J* = 12 Hz, 1H).

2-(Benzyloxycarbonylaminoethyl)-5-acetamido-3,5-dideoxy-D-glycero-α-D-galacto-2-nonulopyranosylonate-(2-3)-β-D-galactopyranosyl-(1→4)-2-acetamido-2-deoxy-β-D-glucopyranoside (3) (Neu5Ac-α2-3-LacNAc-β-Ethyl-NH-Cbz): white solid; yield 68%. ^{1}H-NMR (500 MHz, D_2O) δ = 7.36-7.42 (m, 5H), 5.32 (m, 2), 4.53 (d, *J* = 5 Hz, 1H), 4.50 (d, *J* = 7.7, 1H), 4.12 (dd, *J* = 9.1, 13.3 Hz,1H), 3.81-3.94 (m, 5H), 3.60-3.72 (m, 11H), 3.52-3.62 (m, 3H), 3.26–3.36 (bs, 2H), 2.75 (dd, *J* = 4.9, 11.9 Hz, 1H), 2.03 (s, 3H), 1.96 (s, 3H), 1.80 (t, *J* = 11.9 Hz, 1H).

2-(Benzyloxycarbonylaminoethyl)-5-acetamido-3,5-dideoxy-D-glycero-α-D-galacto-2-nonulopyranosylonate-(2-6)-β-D-galactopyranosyl-(1→4)-2-acetamido-2-deoxy-β-D-glucopyranoside (4) (Neu5Ac-α2-6-LacNAc-β-Ethyl-NHCbz): white solid; yield 59%. ^{1}H-NMR (500 MHz, D_2O) δ = 7.38-7.46 (m, 5H), 5.31 (m, 2), 4.53 (d, *J* = 5 Hz, 1H), 3.84-3.98 (m, 5H), 3.75-3.81 (m, 3H), 3.56-3.73 (m, 10H), 3.51-3.54 (m, 3H), 3.30 (bs, 2H), 2.65 (dd, *J* = 4.9, 11.9 Hz, 1H), 2.05 (s, 3H), 1.98 (s, 3H), 1.69 (t, *J* = 11.9 Hz, 1H).

2-Azidoethyl 5-acetamido-3,5-dideoxy-D-glycero-α-D-galacto-2-nonulopyranosylonate-(2-3)-β-D-galactopyranosyl-(1→4)-2-acetamido-2-deoxy-β-D-glucopyranoside (5) (Neu5Ac-α2-3-LacNAc-β-Ethyl-N_3): white solid; yield 52%. ^{1}H-NMR (500 MHz, D_2O) δ = 4.41 (dd, *J* = 8, 15 Hz, 1H), 4.10 (d, *J* = 7.5, 1H), 3.81-4.05 (m, 4H), 3.81-3.92 (m, 5H), 3.66-3.79 (m, 7H), 3.54-3.68 (m, 8H), 2.75 (dd, *J* = 4.5, 12 Hz, 1H), 2.03 (s, 6H), 1.78 (t, *J* = 12 Hz, 1H).

2-Azidoethyl 5-acetamido-3,5-dideoxy-D-glycero-α-D-galacto-2-nonulopyranosylonate-(2-6)-β-D-galactopyranosyl-(1→4)-2-acetamido-2-deoxy-β-D-glucopyranoside (6) (Neu5Ac-α2-6-LacNAc-β-Ethyl-N_3): white solid; yield 77%. ^{1}H-NMR (500 MHz, D_2O) δ = 4.60 (dd, *J* = 8, 15 Hz, 1H), 4.42 (d, *J* = 7.5, 1H), 3.81-4.05 (m, 4H), 3.94-4.20 (m, 5H), 3.58-3.92 (m, 10H), 3.48-3.51 (m, 5H), 2.65 (dd, *J* = 4.5, 12 Hz, 1H), 2.40 (s, 3H), 2.03 (s, 3H), 1.68 (t, *J* = 12.5 Hz, 1H).

5-acetamido-3,5-dideoxy-*D*-glycero-α-*D*-galacto-2-nonulopyranosylonate-(2→3)-*O*-D-galactopyranosylβ(*1→4*)-2-(acetamido)-2-deoxy-β-D-glucopyranose (7) (Neu5Ac-α2-3LacNAc): white solid; yield 79%. ^{1}H-NMR (500 MHz D_2O) δ = 4.52 (d, *J* = 7.5 Hz, 1H), 4.12 (d, *J* = 7.5, 1H), 3.81-4.05 (m, 9H), 3.52-3.78 (m, 11H), 2.75 (dd, *J* = 3.5, 12.5 Hz, 1H), 2.04 (s, 6H), 1.78 (t, *J* = 12.5 Hz, 1H).

5-acetamido-3,5-dideoxy-*D*-glycero-α-*D*-galacto-2-nonulopyranosylonate-(2→3)-*O*-D-galactopyranosylβ(*1→4*)-2-(acetamido)-2-deoxy-β-D-glucopyranose (8) (Neu5Ac-α2-6-LacNAc): white solid; yield 75%. ^{1}H-NMR (500 MHz, D_2O) δ = 4.52 (d, *J* = 7.5 Hz, 1H), 4.45 (d, *J* = 7.5, 1H), 3.80-4.02 (m, 9H), 3.49-3.75 (m, 11H), 2.68 (dd, *J* = 3.5, 12.0 Hz, 1H), 2.04 (s, 6H), 1.70 (t, *J* = 12.0Hz, 1H).

Copies of ^{1}H NMR and mass spectra

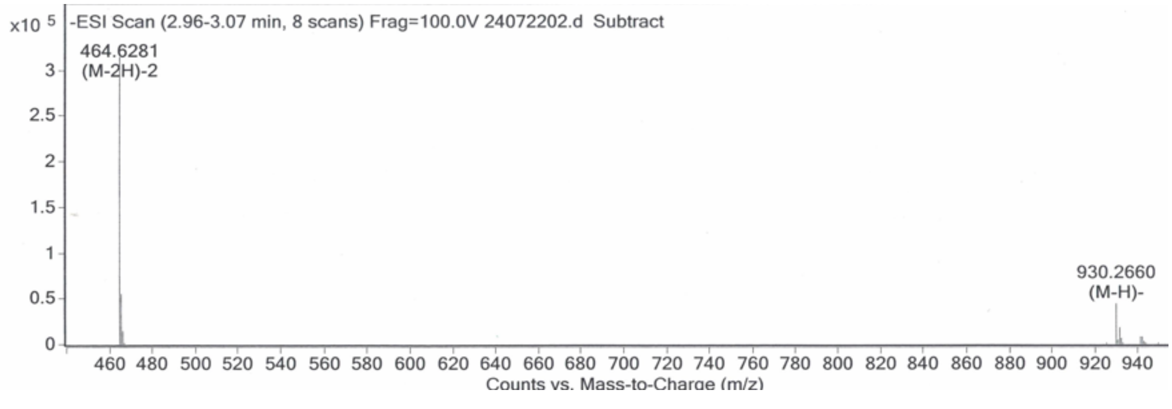

Figure S1. ESI-MS spectrum of compound **1**.

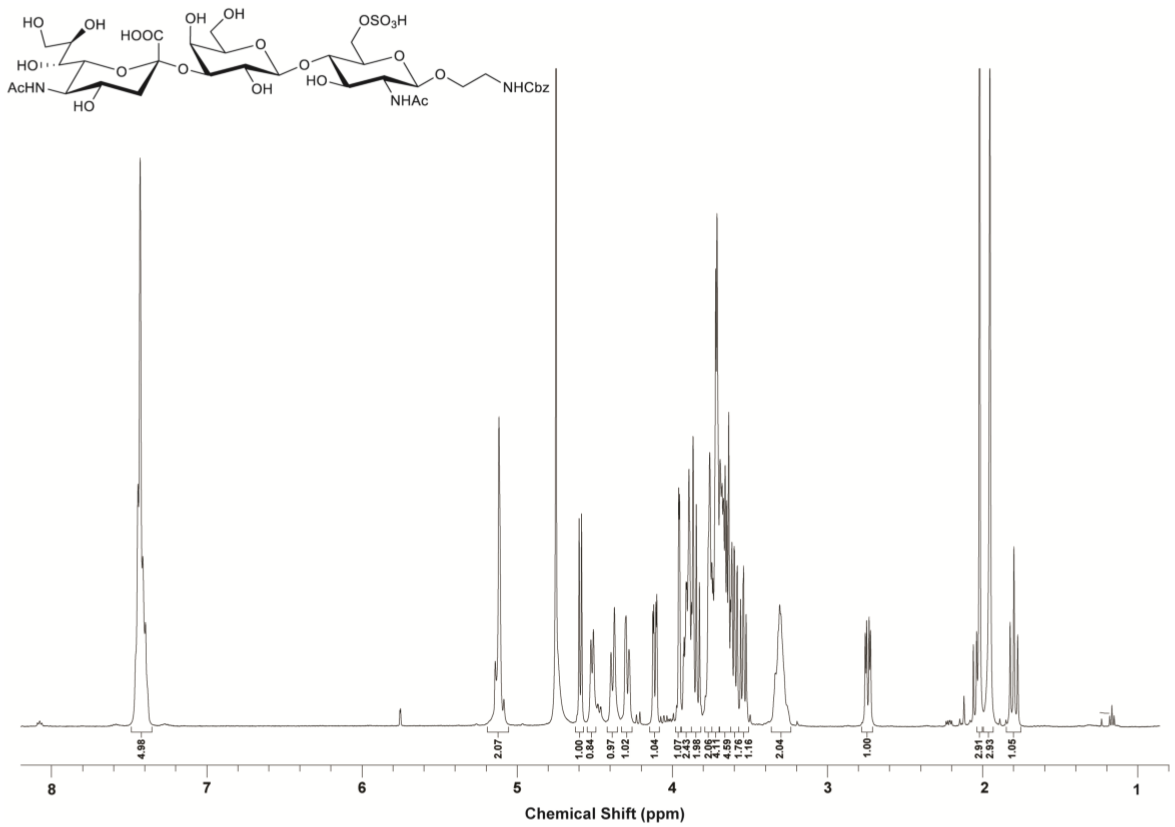

Figure S2. ^{1}H NMR (500 MHz, D_2O) spectrum of compound **1**.

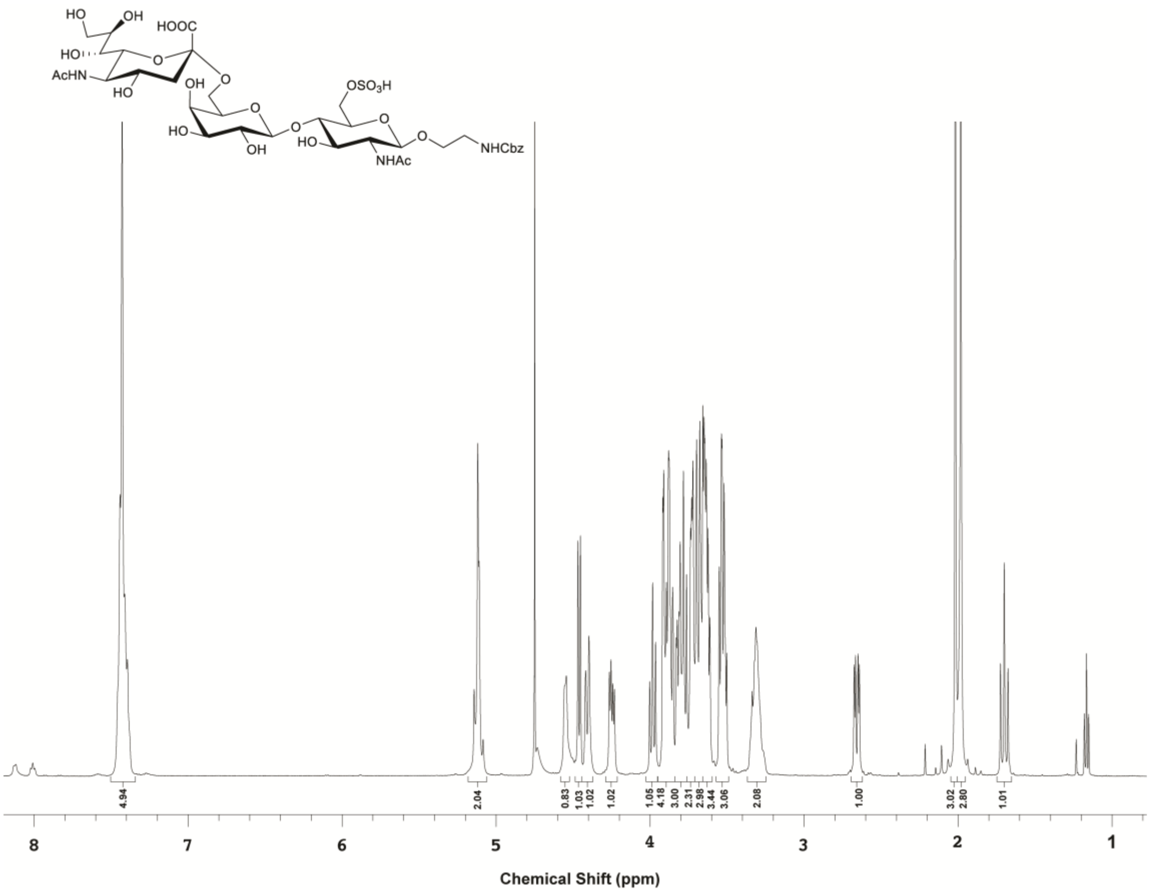

Figure S3. ^{1}H NMR (500 MHz, D_2O) spectrum of compound **2**.

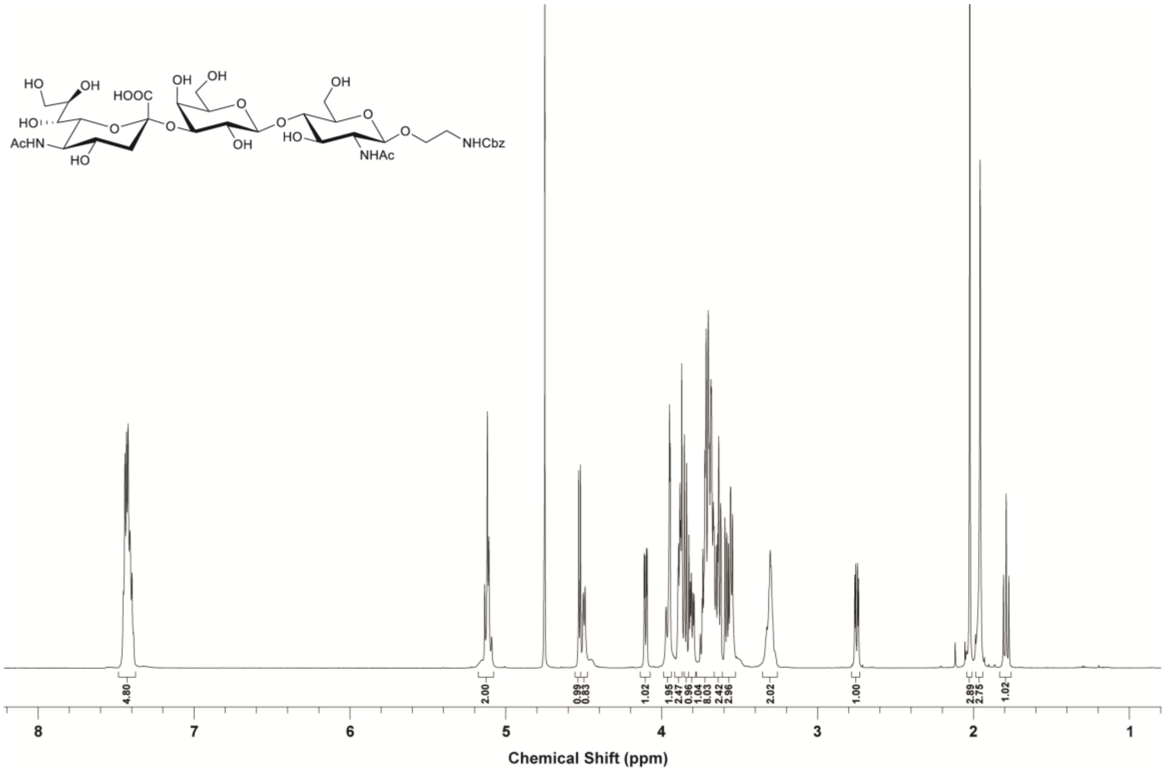

Figure S4. ^{1}H NMR (500 MHz, D_2O) spectrum of compound **3**.

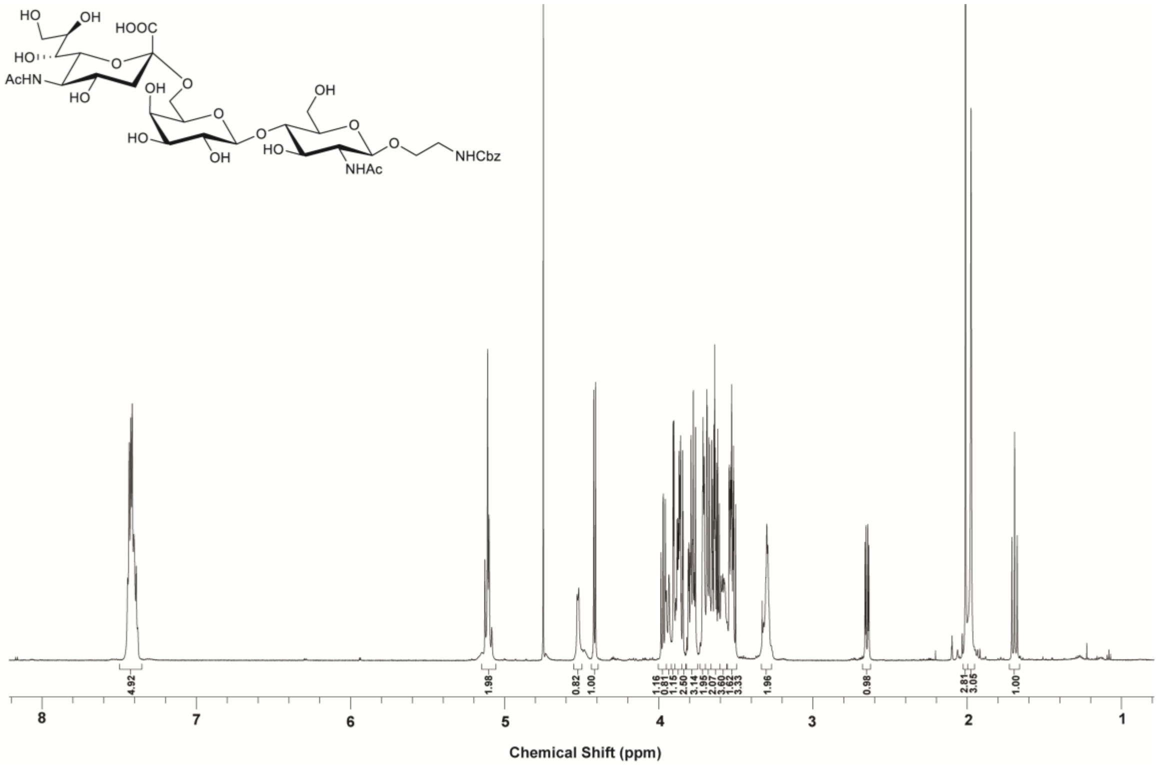

Figure S5. ^{1}H NMR (500 MHz, D_2O) spectrum of compound **4**.

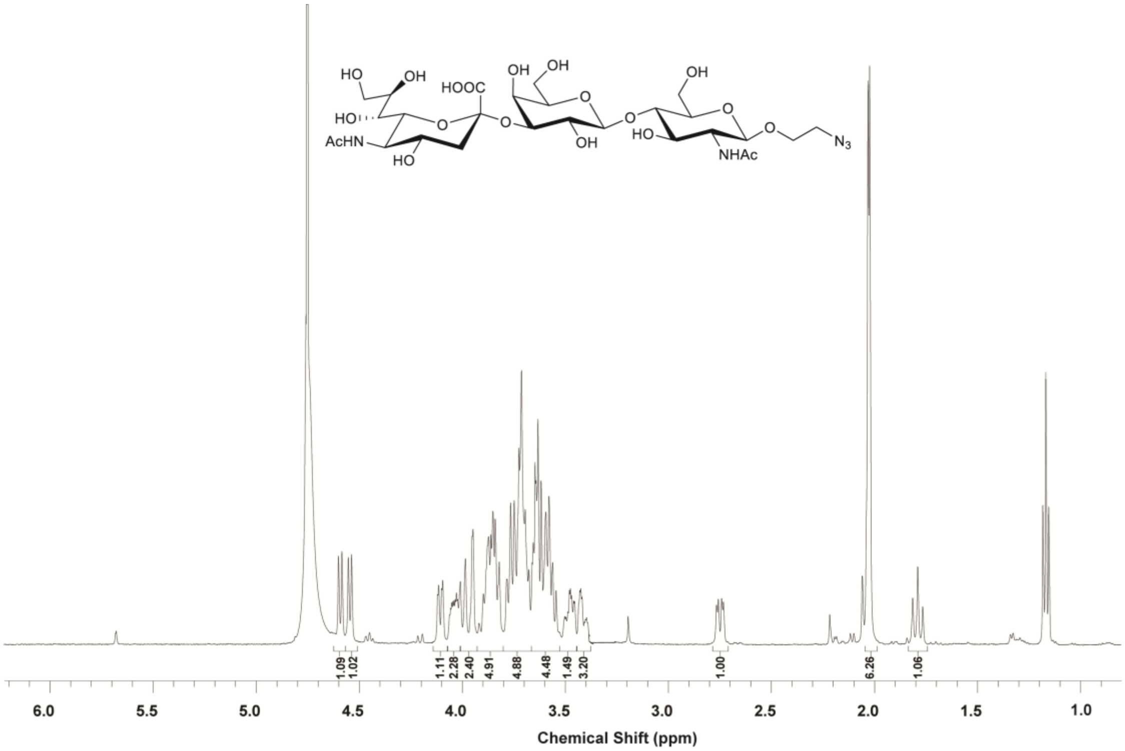

Figure S6. ^{1}H NMR (500 MHz, D_2O) spectrum of compound **5.**

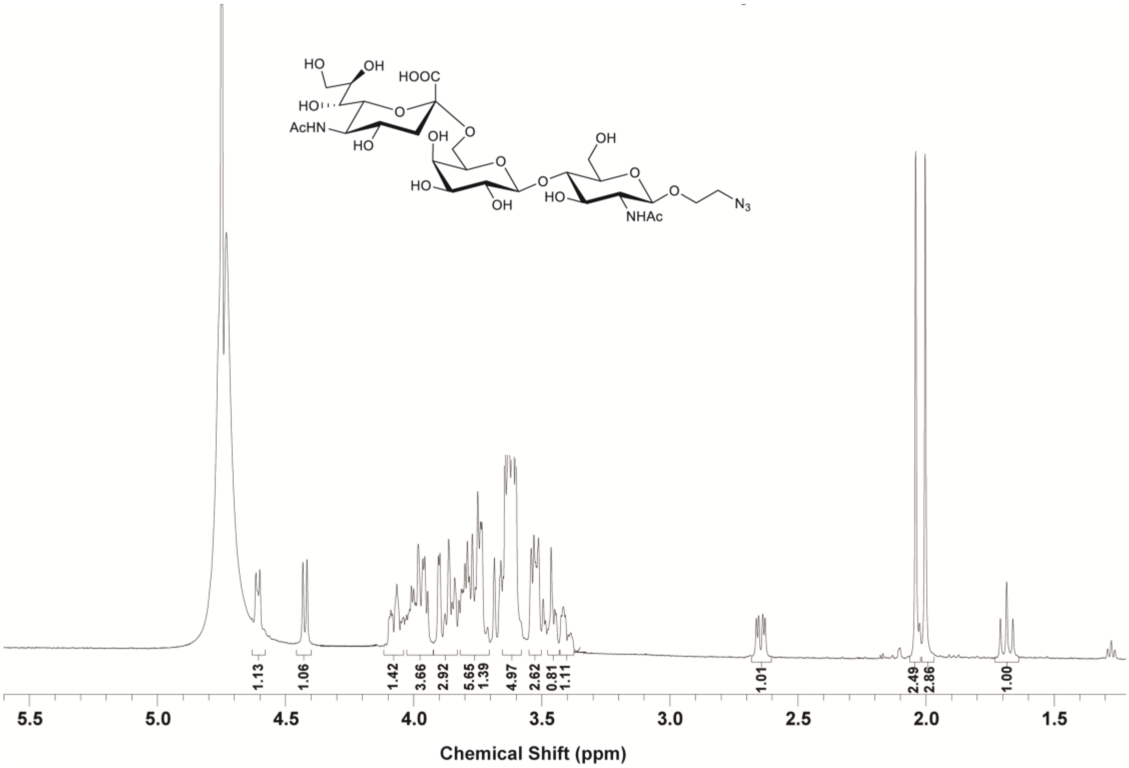

Figure S7. ^{1}H NMR (500 MHz, D_2O) spectrum of compound **6.**

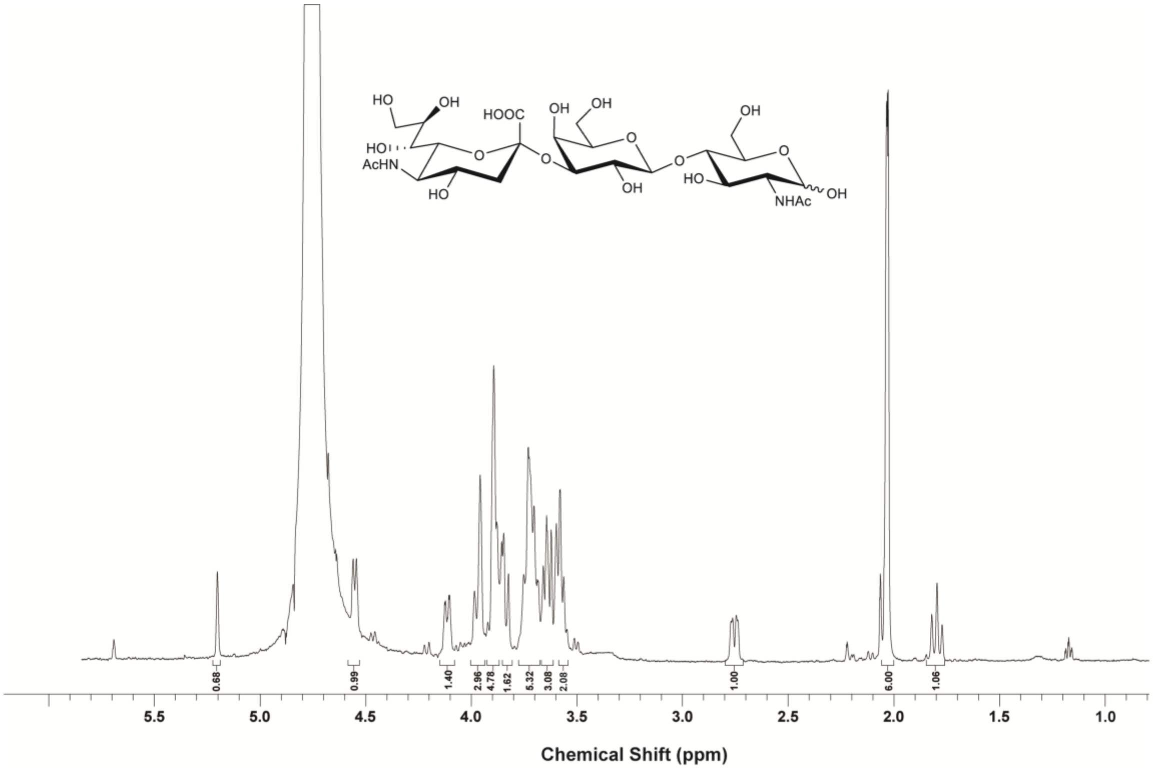

Figure S8. ^{1}H NMR (500 MHz, D_2O) spectrum of compound **7**.

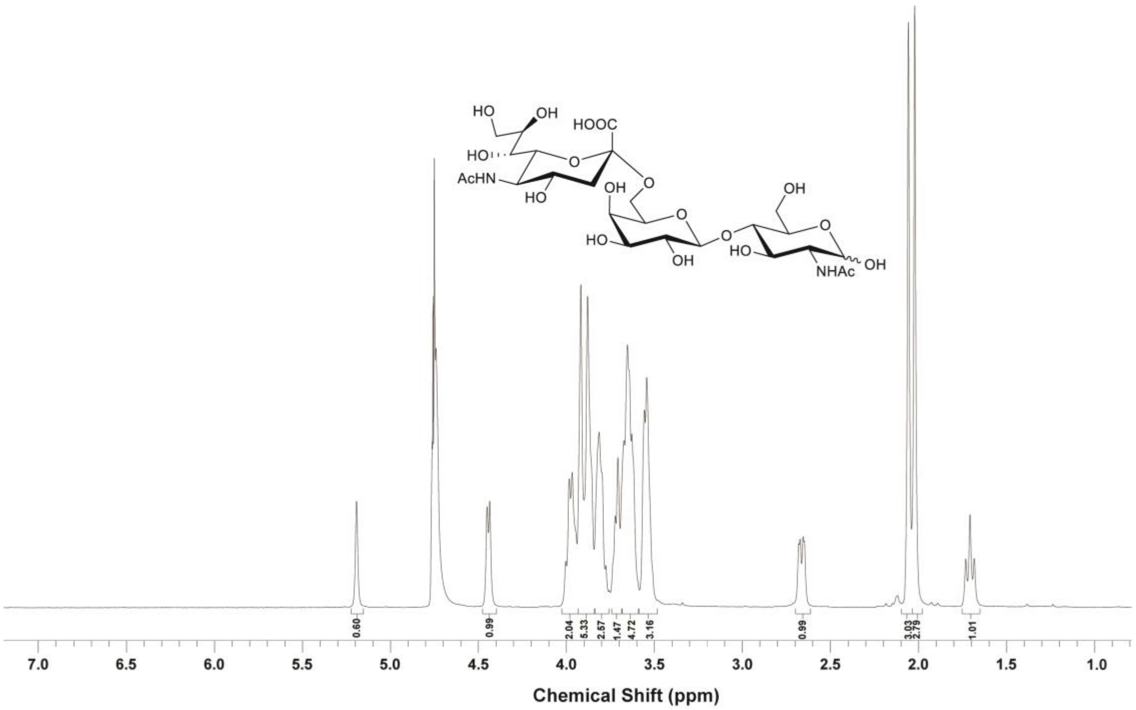

Figure S9. ^{1}H NMR (500 MHz, D_2O) spectrum of compound **8**.

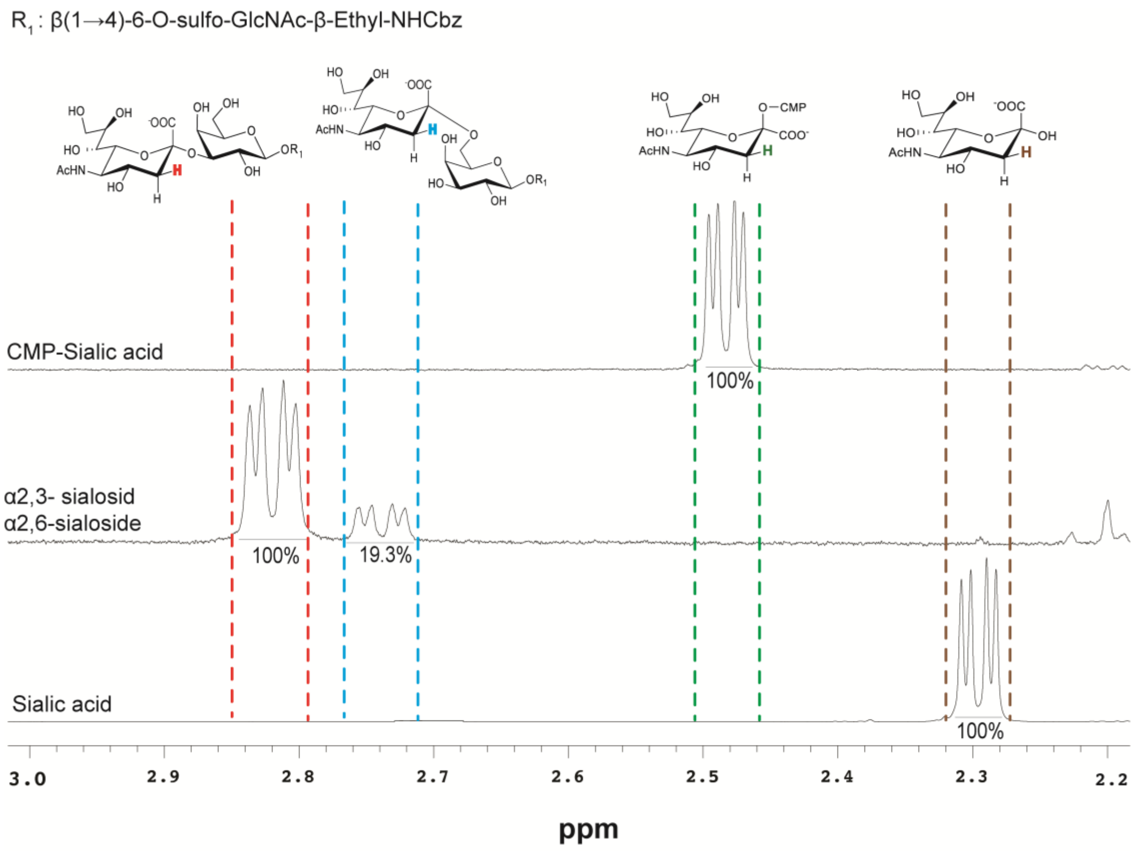

Figure S10: Stacked spectra of, bottom: Sialic acid, middle: mixture of α2-3 and α2-6-sulfated sialoside, and top: CMP-Sialic acid.
